# Supplementary material for: Giant tunnelling electroresistance in atomic-scale ferroelectric tunnel junctions
Source: Nat Commun. 2024 Jan 24;15:693. doi: 10.1038/s41467-024-44927-7 (PMC10808203; doi:10.1038/s41467-024-44927-7)
Supplement: Supplementary file 1 — Supplementary Information [file 41467_2024_44927_MOESM1_ESM.pdf]

Supplementary Information

Giant Tunnelling Electroresistance in Atomic-Scale Ferroelectric Tunnel Junctions

Yueyang Jia[1,†], Qianqian Yang[2,†], Yue-Wen Fang[3,4,*], Yue Lu[5], Maosong Xie[1], Jianyong Wei[1], Jianjun Tian[2], Linxing Zhang[2,*], Rui Yang[1,6,*]

[1]*University of Michigan – Shanghai Jiao Tong University Joint Institute, Shanghai Jiao Tong University, Shanghai 200240, China*
[2]*Beijing Advanced Innovation Center for Materials Genome Engineering, Institute for Advanced Materials and Technology, University of Science and Technology Beijing, Beijing 100083, China*
[3]*Fisika Aplikatua Saila, Gipuzkoako Ingeniaritza Eskola, University of the Basque Country (UPV/EHU), Europa Plaza 1, 20018 Donostia/San Sebastián, Spain*
[4]*Centro de Física de Materiales (CSIC-UPV/EHU), Manuel de Lardizabal Pasealekua 5, 20018 Donostia/San Sebastián, Spain*
[5]*Beijing Key Laboratory of Microstructure and Properties of Solids, Faculty of Materials and Manufacturing, Beijing University of Technology, Beijing 100124, China.*
[6] *State Key Laboratory of Radio Frequency Heterogeneous Integration, Shanghai Jiao Tong University, Shanghai, China*

[†]Equal Contribution.

[*]Corresponding authors. Emails: yuewen.fang@ehu.eus, linxingzhang@ustb.edu.cn, rui.yang@sjtu.edu.cn

Supplementary Notes

1. Transport Mechanism for BSO-based FTJs

(1.1) Direct tunnelling (DT)

The direct tunnelling current through a trapezoidal potential barrier is described as[1]:

$$I_{DT} = A\cdot C\frac{\exp\left\{\alpha(V)\left[\left(\Phi_2-\frac{eV}{2}\right)^{3/2}-\left(\Phi_1+\frac{eV}{2}\right)^{3/2}\right]\right\}}{\alpha^2(V)\left[\left(\Phi_2-\frac{eV}{2}\right)^{1/2}-\left(\Phi_1+\frac{eV}{2}\right)^{1/2}\right]^2}\times\sinh\left\{\frac{3}{2}\alpha(V)\left[\left(\Phi_2-\frac{eV}{2}\right)^{1/2}-\left(\Phi_1+\frac{eV}{2}\right)^{1/2}\right]\frac{eV}{2}\right\}, \quad \text{(S1)}$$

$$C = -(4em_n^*)/(9\pi^2\hbar^3)\,, \quad \text{(S2)}$$

$$\alpha(V) = [4d(2m_n^*)^{1/2}]/[3\hbar(\Phi_1-\Phi_2+eV)]\,, \quad \text{(S3)}$$

where A is the device lateral size, $\Phi_{1,2}$ is the barrier height at the metal/ferroelectric (Cr/BSO) and ferroelectric/semiconductor (BSO/NSTO) interface, m_n^* is the effective mass of electrons in NSTO, $\hbar$ is the reduced Planck constant, and d is the BSO thickness.

(1.2) Thermally assisted tunnelling

The thermally assisted tunnelling current is[2]:

$$I = A\cdot J_0\exp(qV/E_0)\,, \quad \text{(S4)}$$

where q is the electron charge, V is the applied voltage, E_0 is a temperature-dependent energy given by:

$$E_0 = nk_BT\,, \quad \text{(S5)}$$

where n is the ideality factor which equals to unity for ideal thermionic emission over a Schottky-type barrier, k_B is Boltzmann constant, and T is temperature.

The temperature-dependent saturation current J_0 in Supplementary equation (4) is given by:

$$J_0(T) = \frac{A^*T^2\pi^{1/2}E_{00}^{\ 1/2}\left[q\left(V_{bi}-V\right)+\phi_n\right]^{1/2}}{k_BT\cosh(E_{00}/k_BT)}\times\exp\left(\frac{\varphi_n}{k_BT}-\frac{qV_{bi}+\phi_n}{E_0}\right), \quad \text{(S6)}$$

$$E_{00} = \frac{qh}{4\pi}\left(\frac{N_D}{m^* \varepsilon_r(T)\varepsilon_0}\right)^{1/2}, \tag{S7}$$

where V_{bi} is the built-in potential of the Schottky-like barrier due to the surface depletion, $\varepsilon_r(T)$ is the temperature-dependent relative permittivity of NSTO which can be described by the Barrett's formula[3]:

$$\varepsilon_r(T) = \frac{1635}{\coth\left(\frac{44.1}{T}\right) - 0.937}. \tag{S8}$$

ϕ_n is the difference between the conduction band minimum (E_C) and the Fermi level of NSTO (E_F), which can be estimated using the effective mass of electrons (m_n^*) in NSTO[4]:

$$\phi_n = \frac{E_F - E_C}{q} \approx \frac{k_B T}{q}\left[\ln\left(\frac{N_D}{N_C}\right) + 2^{-3/2}\left(\frac{N_D}{N_C}\right)\right], \tag{S9}$$

where N_D is the doping concentration, N_C is the density of states (DOS) close to E_C of NSTO given by:

$$N_C = 2\left(\frac{2\pi m_n^* k_B T}{h^2}\right)^{3/2}. \tag{S10}$$

The effective Richardson constant of NSTO in Schottky junction is 156 A/(K^2cm^2), and $m_n^* = 1.3\, m_0$.[5] The electron affinity of NSTO is 4.08 eV.

(1.3) Discussion on theoretical investigations of transport properties

Although the tunnelling mechanism in the ferroelectric tunnel junctions employing doped semiconducting electrodes as in this work has been elucidated in a former study by Shen et al[6], further theoretical investigations based on density functional non-equilibrium Green's function methods are worth carrying out to supplement the tunnelling model for fully understanding the transport properties of our studied ferroelectric tunnel junctions.

2. Energy Barrier in NSTO Depletion Region.

When the Au/Cr/BSO/BSTO FTJs are in HRS, the depletion region and extra energy barrier at NSTO surface are formed. Thermally assisted tunnelling exists, leading to an ideality factor $n>1$. In this case, $n = 1+C_d/C_f$, where C_d and C_f are the high-frequency capacitances of the depletion region in NSTO and the ferroelectric barrier in the BSO layer, respectively[7,8]. The build-in potential of a typical Schottky-like junction can be obtained through capacitance–voltage (C–V) measurement. For a semiconductor depletion region, the capacitance is calculated as[4]:

$$C_d = \left(\frac{q\varepsilon_r N_D A^2}{2\,(V_{bi}-V_d)} \right)^{1/2}, \tag{S11}$$

where V_d is the reverse-bias voltage that drops across the depletion region. The equation can be rewritten as:

$$\left(\frac{1}{C_d} \right)^2 = \frac{2\,(V_{bi}-V_d)}{q\varepsilon_0\varepsilon_r N_D A^2} \ . \tag{S12}$$

It can be found that the C_d^{-2} shows a linear dependent on V_d. Therefore, the doping concentration of NSTO can be calculated from the slope and the build-in potential V_{bi} can be extracted from the intercept. Besides, the depletion region width W_d can be estimated based on the extracted V_{bi} and N_D:

$$W_d = \left(\frac{2\varepsilon_0\varepsilon_r V_{bi}}{qN_D} \right)^{1/2} . \tag{S13}$$

In our metal/ferroelectric/semiconductor FTJs, the capacitance C_f for the BSO layer and the capacitance C_d for the space charge region are connected in series. The total capacitance C can then be obtained from $1/C = 1/C_d + 1/C_f = n/C_d$. The reverse-bias voltage that drops across the depletion region (V_d) is then V/n, where V is the total reversed biased voltage applied on the whole junction. By linearly fitting to the $C_d^{-2} - V_d$ plots, the doping concentration of NSTO and the build-in potential V_{bi} can be obtained (Supplementary Fig. 18), and the depletion width in the BSO/NSTO interface is calculated.

3. Evaluation of Nonlinearity for Potentiation and Depression Processes

The conductance (G) update process with pulse number (N) for long-term potentiation (LTP) and long-term depression (LTD) is given by Supplementary equations (14–16)[9]:

$$G_{LTP} = G_{\min} + G_0\left(1 - \exp\left(-\frac{N}{A_p}\right)\right), \tag{S14}$$

$$G_{LTD} = G_{\max} - G_0\left(1 - \exp\left(-\frac{N - N_{\max}}{A_d}\right)\right), \tag{S15}$$

$$G_0 = \frac{G_{\max} - G_{\min}}{1 - \exp\left(-N_{\max}/A_{p/d}\right)}\ , \tag{S16}$$

where $N_{\max}$, $G_{\min}$, and $G_{\max}$ represent the maximum pulse number, minimum conductance, and maximum conductance values, respectively. A_p and A_d are the fitting parameters related to the nonlinearity of the potentiation and depression processes, respectively.

The nonlinearity factor (α) of conductance for potentiation (α_p) and depression (α_d) can be calculated from:

$$\alpha_{p/d} = \frac{1.727}{A_{p/d} + 0.162} \tag{S17}$$

By fitting to the experimentally measured conductance *vs*. pulse number data using Supplementary equations (14) or (15), the values of $A_{p/d}$ can be extracted, and the nonlinearity factor α is then obtained. The curve will be more linear when α is much closer to zero.

Supplementary Figures

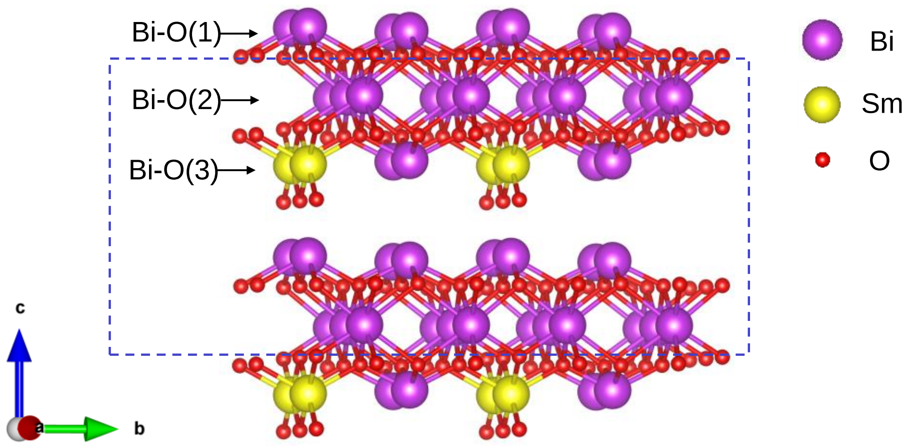

Figure S1. The atomic model of BSO thin film, with the dashed blue box showing the BSO film illustrated in Fig. 1b. In the grown BSO film, some regions have three Bi-O layers, while some regions may have four Bi-O layers. In regions with three Bi-O layers, the thickness is ~0.9 nm; and in regions with four Bi-O layers, the thickness can reach ~1.1 nm. The X-ray reflectivity measurements and the fitting results confirm that the thickness of the BSO film is 1 nm with a surface roughness of 0.173 nm.

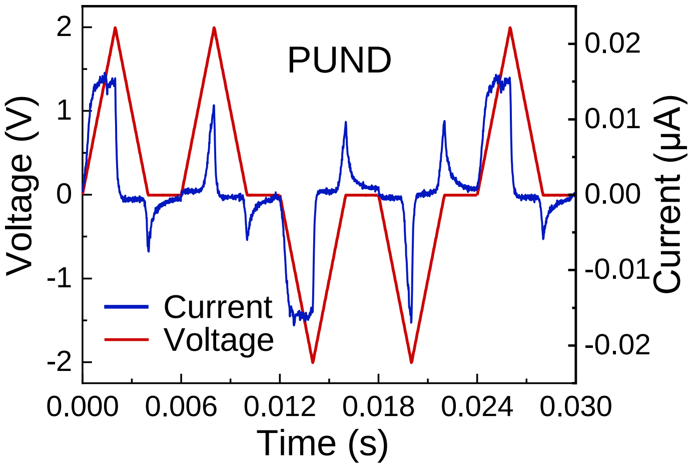

Figure S2. Positive-up-negative-down (PUND) measurements of a FTJ with 1-nm-thick BSO film.

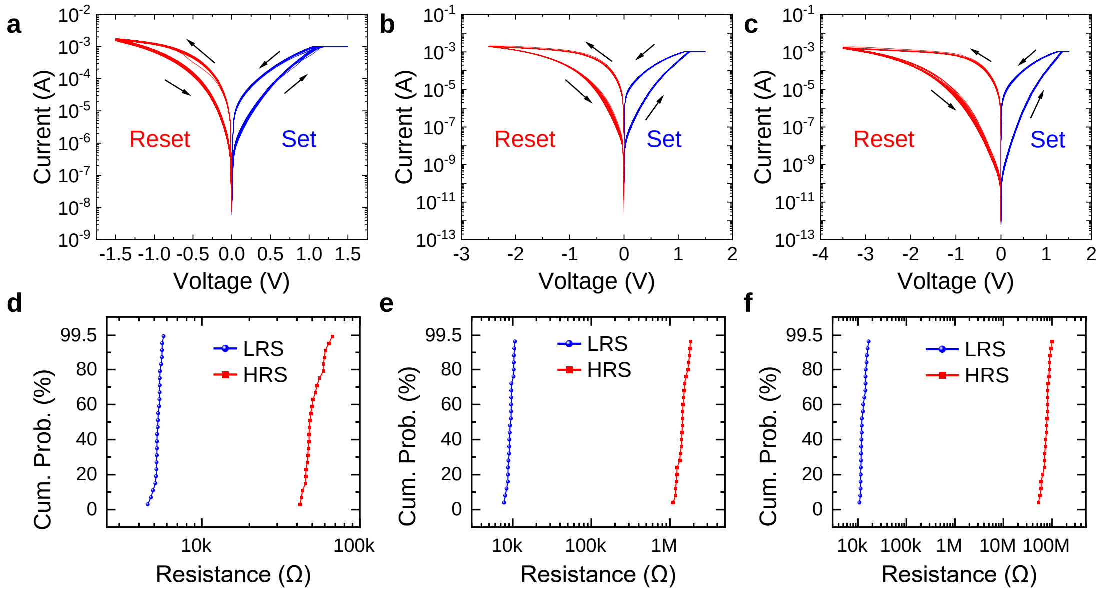

Figure S3. *I*–*V* sweeps with different reset stop voltages and the corresponding resistance distribution for 1-nm-BSO-based FTJ. a–c, The switching characteristics of the FTJ are switched under +1.5 V set voltage and **a** -1.5 V**, b** -2.5 V, and **c** -3.5 V reset stop voltages. Under each measurement condition, the measurements are performed for multiple cycles, showing repeatable *I*–*V* sweeps. The compliance current of 1 mA is applied in the set process to prevent the large tunnelling current from affecting the device performance. **d–f,** The corresponding resistance distribution for measurements in **a**–**c**, respectively. As the reset voltage increases, the HRS resistances increase a lot, and larger TER values are obtained.

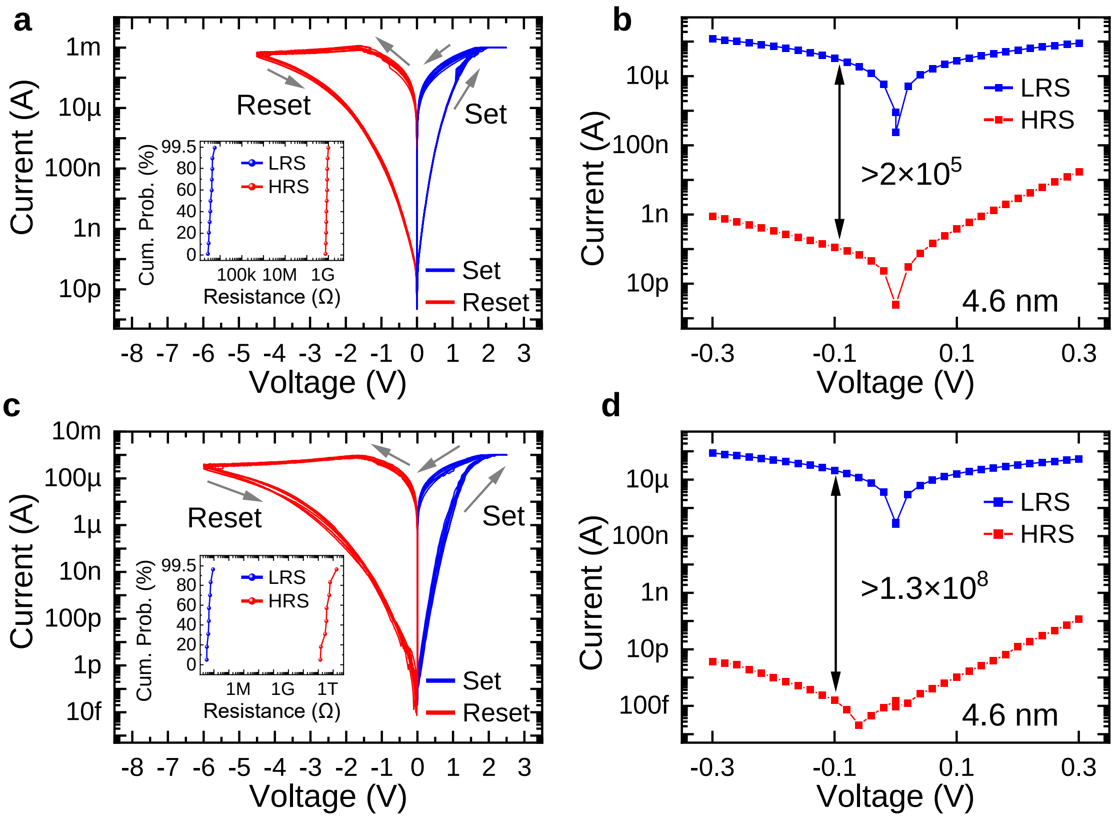

Figure S4. *I–V* sweeps with different reset stop voltages and the corresponding resistance distributions for 4.6-nm-BSO-based FTJ. a, The switching characteristics of the FTJ are switched under +2.5 V set voltage and -4.5 V reset stop voltages. Inset: resistance distribution. **b,** TER over 2×10^5 achieved with -0.1 V read voltage. **c,** The 4.6 nm BSO based FTJ are switched under +2.5 V and -6 V reset stop voltages. Inset: resistance distribution. **d,** over 1.3×10^8 TER is achieved with -0.1 V read voltage. As the reset voltage increases, the HRS resistances increase a lot and larger TER values are obtained, and over 1.2×10^9 TER value with -7 V reset voltage is obtained (Fig. 2d).

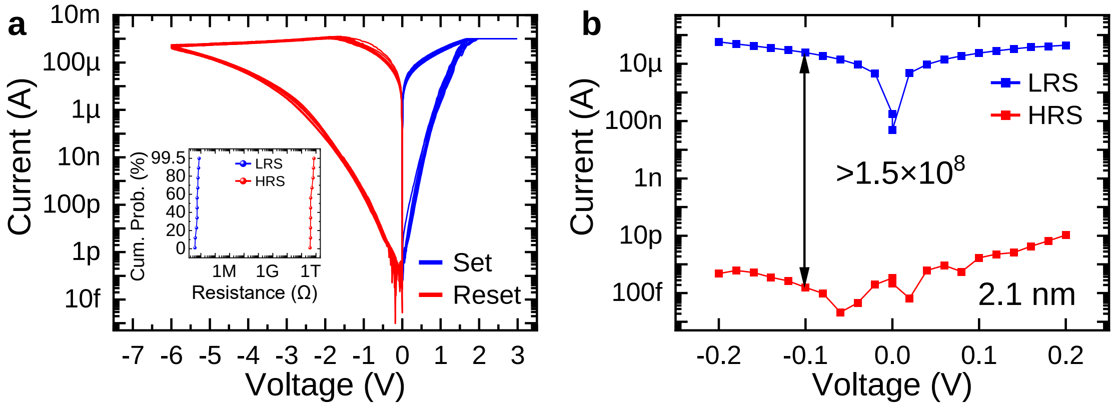

Figure S5. Giant TER in 2.1-nm-BSO-based FTJ. a, *I*–*V* sweep of the FTJ device with BSO thickness of 1 nm. Inset: resistance distribution of low resistance state and high resistance state. **b,** TER over 1.5×10^8 is achieved with -0.1 V read voltage.

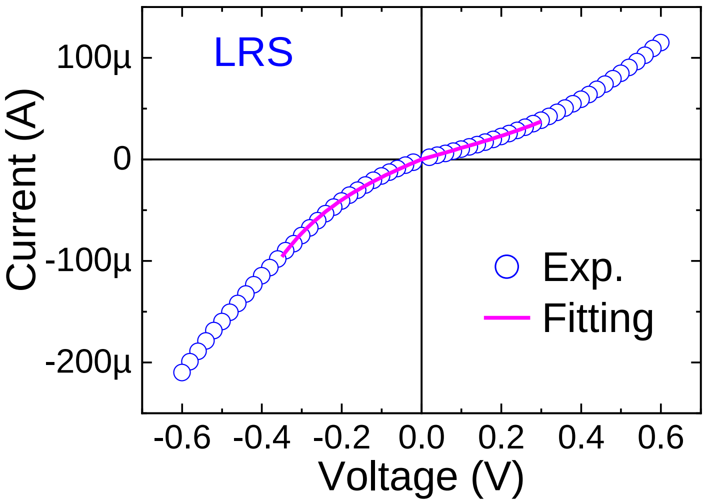

Figure S6. Fitting to the measured *I*–*V* curve for a FTJ in LRS. The *I*–*V* curve at low voltage can be well fitted by the direct tunnelling model. The barrier height at the metal/ferroelectric (Cr/BSO) and ferroelectric/semiconductor (BSO/NSTO) interface are extracted as 1.92 eV and 0.25 eV, respectively.

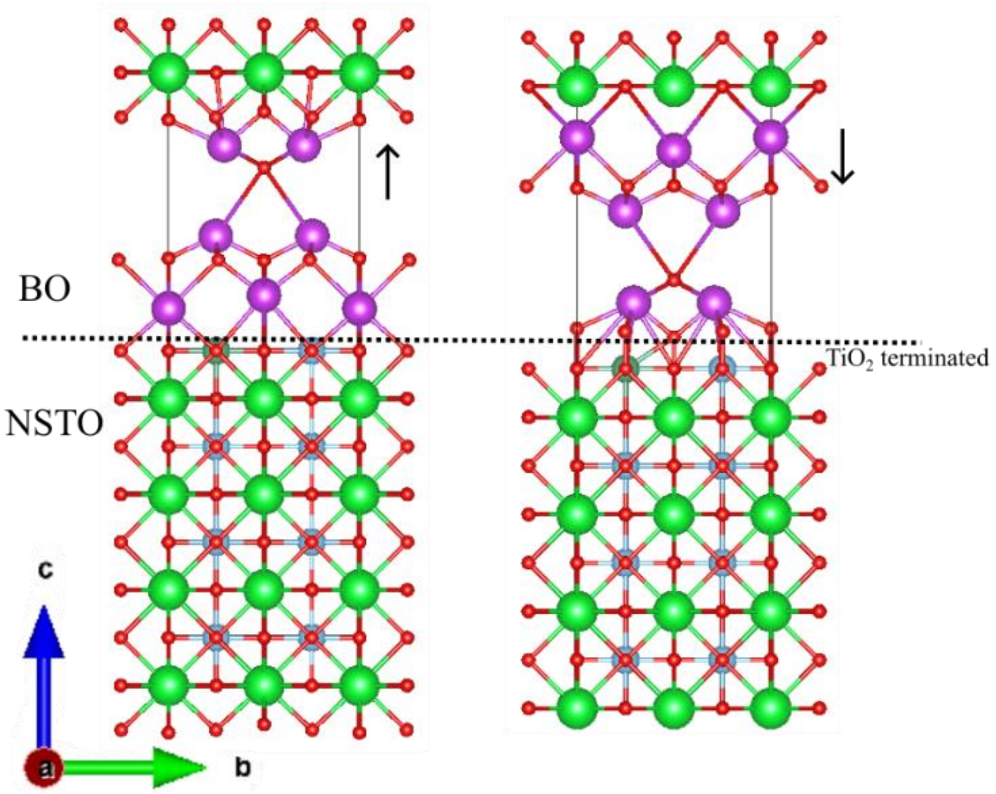

Figure S7. The supercell structure of the interface between bismuth oxide and NSTO. **a,** ‘up’ polarization; **b,** ‘down’ polarization. The polarization switching in the ferroelectric layer is simply simulated by rotating the structure of ferroelectric layer by 180 degrees, which is just an approximated model of the actual heterostructure. This relatively approximated interface model was used just to model the major impact of the ferroelectric polarization switching on the electronic properties of the interface layers. The NSTO has a TiO_2 termination that is energetically favored. The dashed line indicates the interface between the two different components. To reduce the computational difficulty, Sm is not included in this model. It should be mentioned that the ferroelectricity mainly comes from the bismuth atoms, and the role of Sm is not crucial to the interfacial states (evidenced by Supplementary Fig. 8).

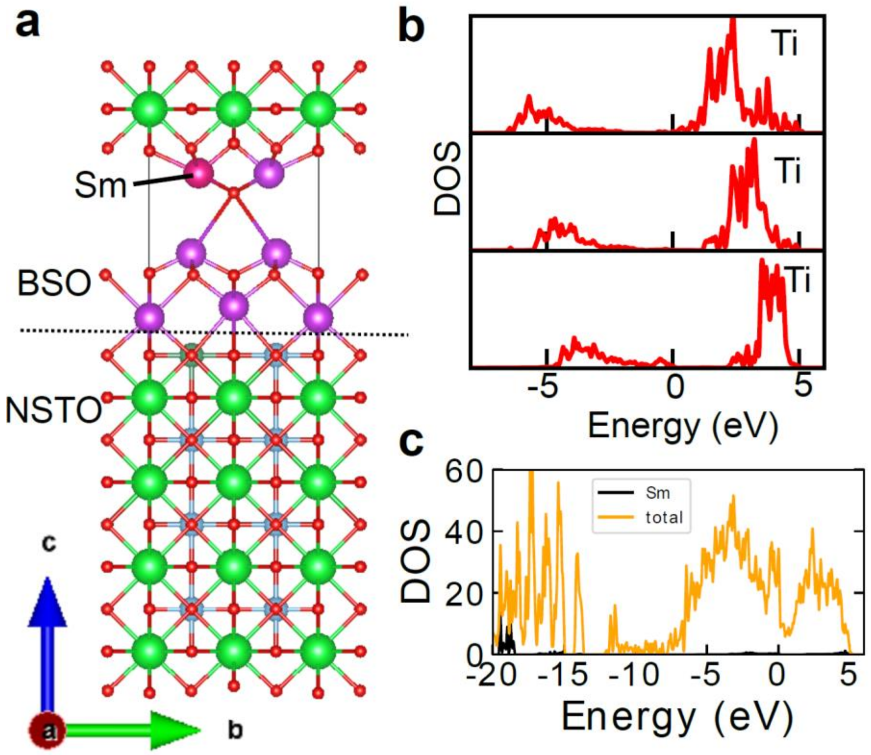

Figure S8: The DFT results of the BSO/NSTO interface. a, Interface structure of BSO/NSTO; **b,** Interface states of Ti, calculated using the Hubbard *U* of 6 eV and *J* of 0.855 eV for Sm. **c,** The comparison between Sm DOS and total DOS, showing that the states near the Fermi level are very weak, and the dominating electronic states of Sm lie as deep as around -20 eV. Therefore, excluding the Sm will not strongly affect the electronic properties at the interface**.**

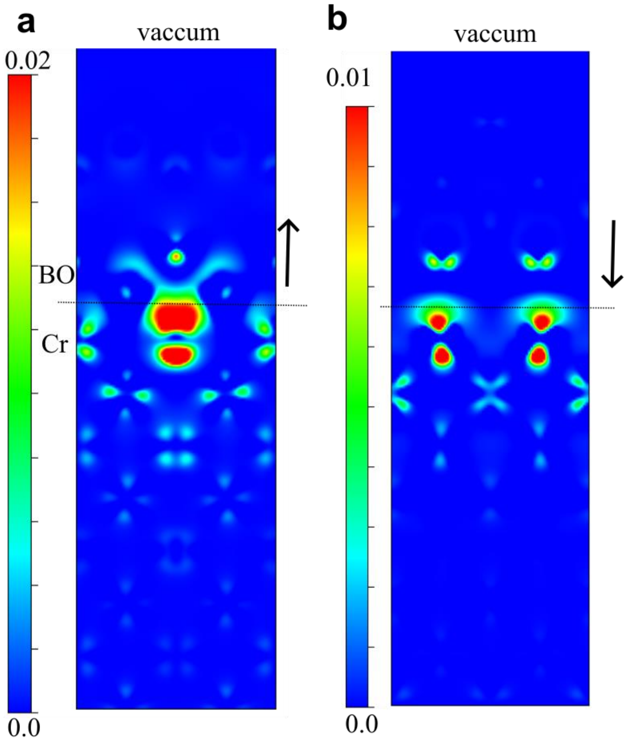

Figure S9: Charge density difference at the interface between ferroelectric bismuth oxide and metallic chromium layers, with the bismuth oxide layer showing (a) upward, and (b) downward polarizations.

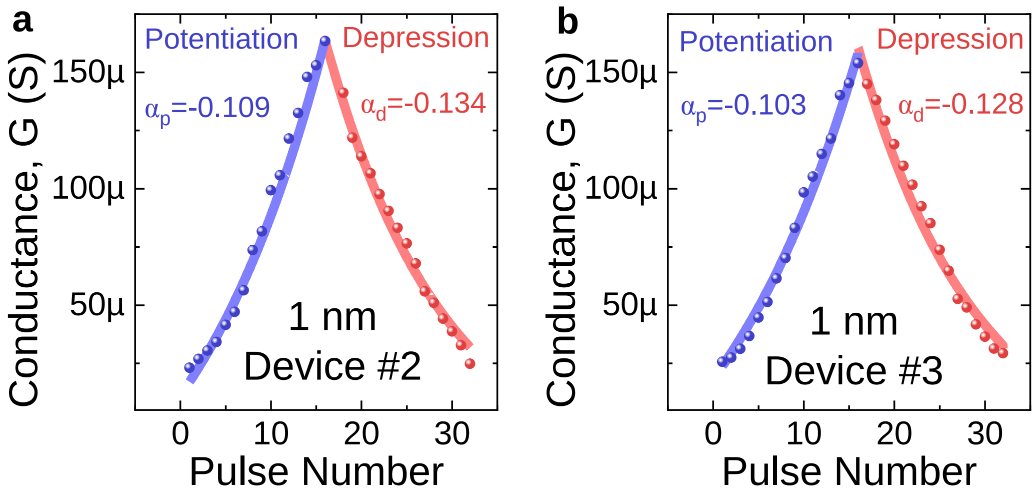

Figure S10. The conductance modulation using the incremental step pulse programming (ISPP) technique, for another two FTJs with 1-nm-thick BSO film. a, Measurement results for FTJ Device #2. **b,** Measurement results for FTJ Device #3. The programming conditions are the same as that in Fig. 3d in the Main Text. The nonlinearities for potentiation (α_p) are extracted as -0.109 and -0.103, and the nonlinearity for depression (α_d) are extracted as -0.134 and -0.128, for Device #2 and Device #3, respectively, showing consistent linear conductance tuning properties among different devices.

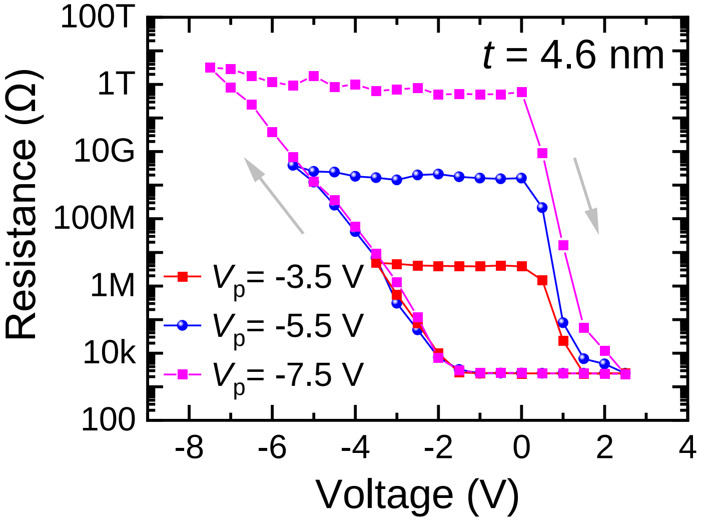

Figure S11. The measured *R*–*V* loops of a FTJ with 4.6-nm-thick BSO layer. Using the ISPP programming method, by gradually increasing the pulse amplitude (V_P), the resistance tuning range is highly enlarged.

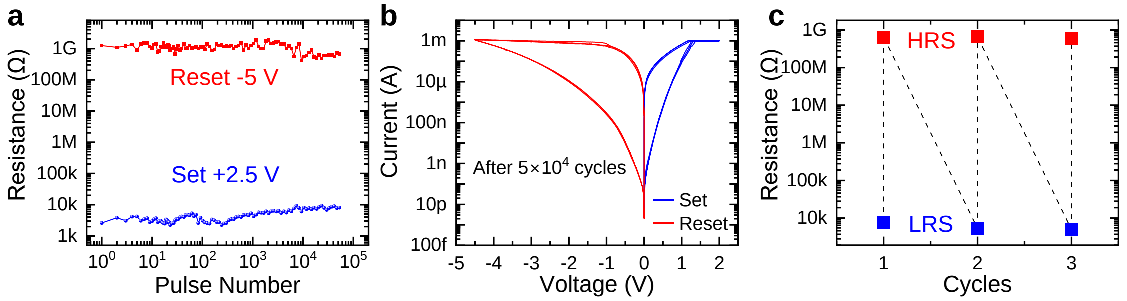

Figure S12. The endurance properties of 1-nm-BSO-based FTJ with TER>10^5**. a,** The endurance measured under +2.5 V set pulses and -5 V reset pulses for 5×10^4 cycles. The device can sustain TER over 10^5. **b,** *I–V* switching curves measured after 5×10^4 endurance cycles, showing no significant degradation. **c,** The extracted resistance from **b**, showing that the device can still maintain TER over 10^5 after 5×10^4 endurance cycles.

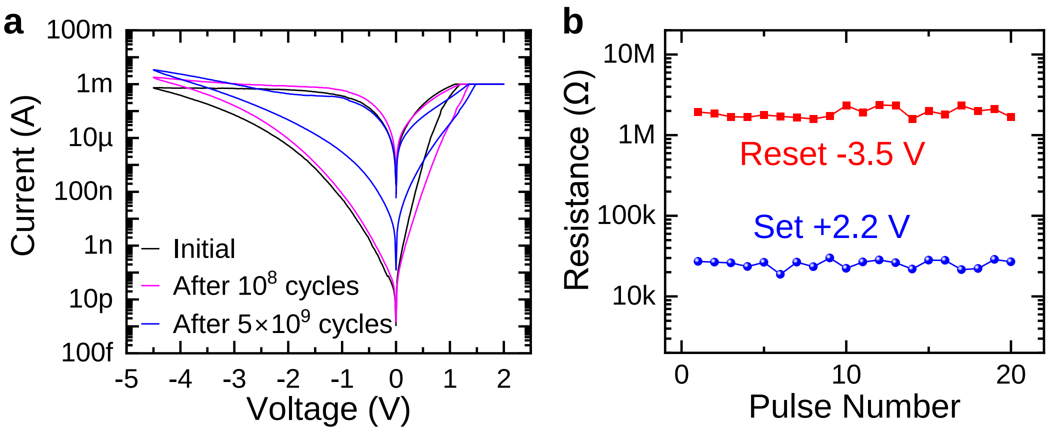

Figure S13. The measured endurance properties of the 1-nm-BSO-based FTJ. a, The *I*–*V* switching characteristics before the endurance measurement and after 10^8 and 5×10^9 endurance cycles. There is no significant degradation of the device performance after 10^8 endurance cycles, while the resistance window shrinks after 5×10^9 endurance cycles. **b,** The HRS and LRS resistances after 5×10^9 endurance cycles, showing that the resistance window degrades under the same programming voltage pulses shown in Fig. 5a in the Main Text. The resistance window can be recovered by slightly increasing the programming voltage amplitudes (Fig. 5b in the Main Text).

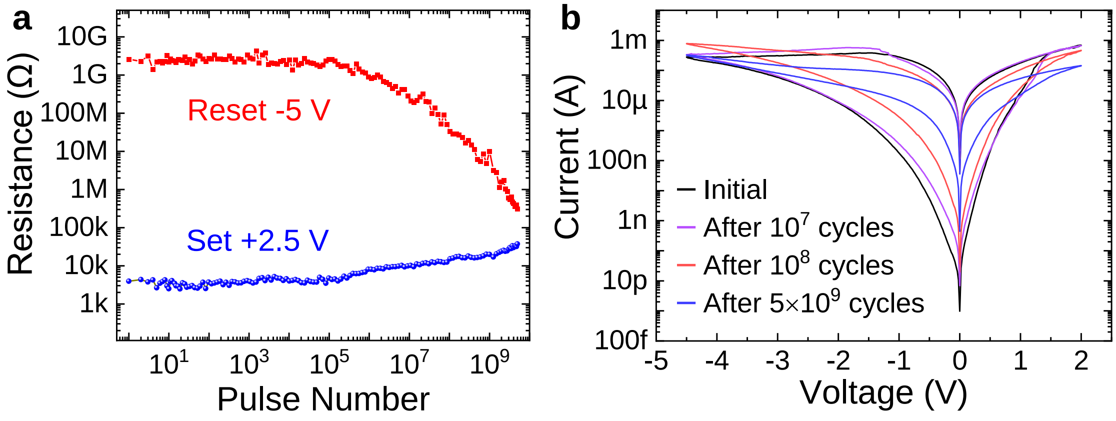

Figure S14. The endurance measurement of 1-nm-BSO-based FTJs with +2.5 V and -5 V programming pulse voltages. **a,** The endurance measured for 5×10^9 cycles. The device can sustain a TER over 10^5 for more than 10^6 cycles, with a total endurance cycle over 5×10^9. **b,** The *I*–*V* switching characteristics measured during the endurance test. The device performance slightly degrades after 10^7 endurance cycles, and the switching window further shrinks as the endurance cycle further increases.

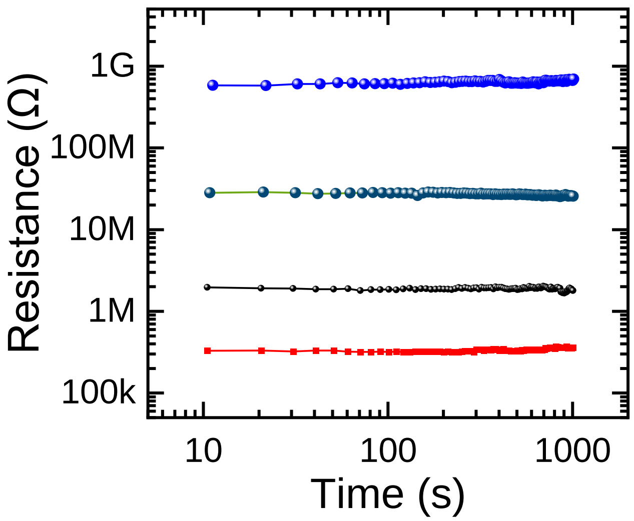

Figure S15. The retention properties of 1-nm-BSO-based FTJ for 4 typical intermediate resistance states. There is no significant shift of the resistance levels, which is consistent with the trend shown in Fig. 5c in the Main Text.

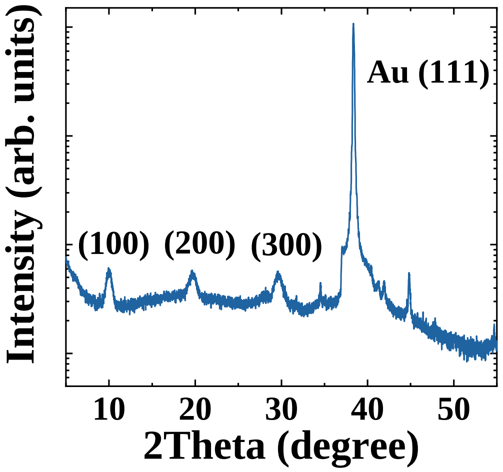

Figure S16. The XRD pattern of the BSO film grown on (111) Au/SiO_2/Si substrate.

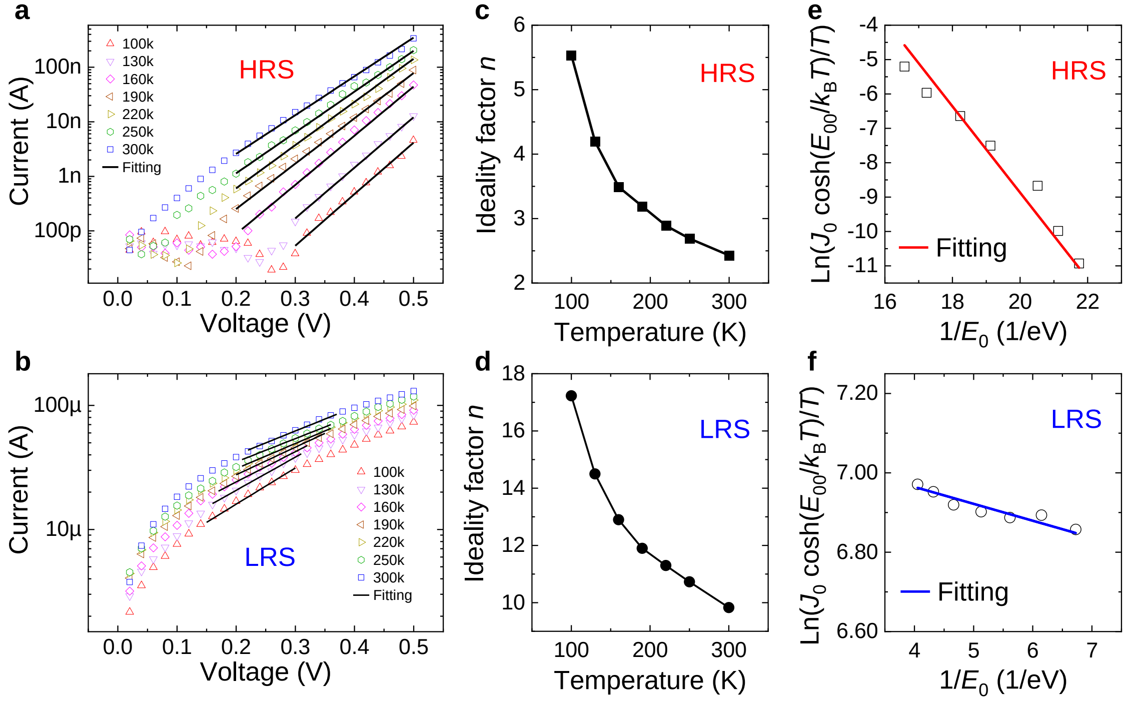

Figure S17. Temperature-dependent measurement and analysis of the 1-nm-thick BSO-based FTJ in both HRS and LRS. **a–b**, The measured *I–V* curves in **a** HRS and **b** LRS at different temperatures ranging from 100K to 300K, with the solid lines fitted by Supplementary equation (4). **c–d,** The extracted ideality factor *n* at different temperature in **c** HRS and **d** LRS. **e–f** The ln (J_0 cosh(E_{00}/k_BT)/T) *vs.* 1/E_0 plots for **e** HRS and **f** LRS, fitted by the Supplementary equation (6), from which V_{bi} of 1.25 V in HRS and 0.1 V in LRS are extracted.

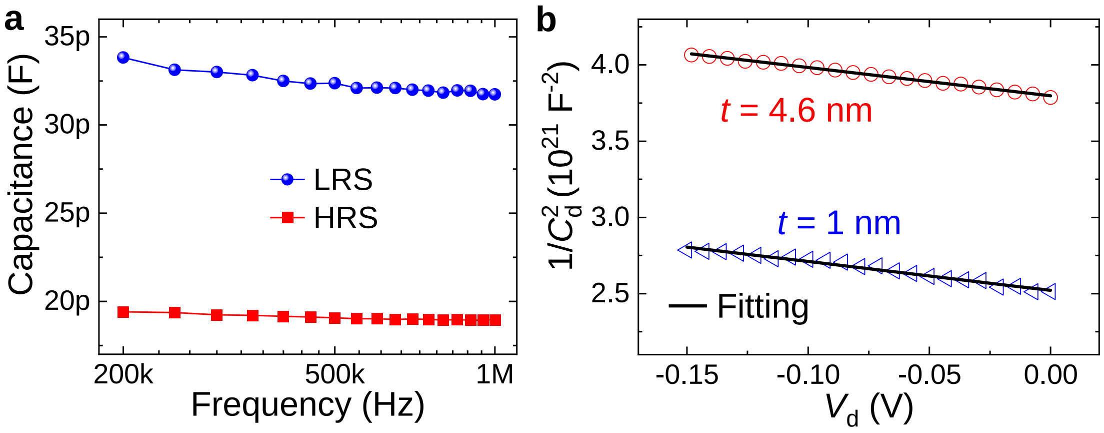

Figure S18. The measured *C*–*V* properties of BSO-based FTJs. a, The capacitance of BSO-based FTJ measured in LRS and HRS at different frequencies, showing that the depletion region form an extra capacitance in series with the ferroelectric capacitance in HRS. **b,** The measured C_d^{-2}–V_d characteristics (symbols) for the HRS of 1-nm-thick and 4.6-nm-thick BSO-based FTJs, with the solid lines showing the fitting to $C_d^{-2}=2(V_{bi}-V_d)/q\varepsilon_0\varepsilon_r N_D A^2$. For FTJs with 1-nm-thick BSO, the V_{bi} is extracted to be 1.35 V, and the corresponding depletion width (W_d) is 9.72 nm. For FTJs with 4.6-nm-thick BSO, the V_{bi} is extracted as 2.04 V, and the corresponding W_d = 11.8 nm.

Supplementary Tables

Table S1. The coercive fields and remanent polarizations of BSO films with different thicknesses.

Thickness (nm)	Coercive Field (MV/mm)	Remanent Polarization($\mu C/cm^2$)
1	1	16.6
2.1	0.95	38
4.6	0.75	50

Table S2. Comparation of BSO-based FTJs with other types of ultra-thin FTJs.

Device Structure	t_{FE}	TER	$V_{Program}$	Retention	Endurance
Au/Cr/CIPS/NSTO[10]	~ 4 nm	$>10^7$	7 V	>10 years	$>10^6$
Pt/SBFO/NSTO[11]	~ 3 nm	$>10^5$	16 V	>10 years	$>10^7$
Pt/BTO/NSTO[12]	~ 2.8 nm	$>10^4$	4 V	>10 years	$>10^4$
Pt/BTO/NSTO[13]	~ 2.8 nm	$>10^7$	7 V	$>10^5$	$>10^5$
Ag/BTO/NSTO[14]	~ 2.4 nm	$>10^2$	3 V	>10 years	$>10^8$
Pt/BTO/NSTO[15]	~ 2 nm	$>10^6$	7 V	---	$>10^7$
Pt/BTO/NSTO[16]	~ 1.6 nm	$>10^6$	7 V	$>10^5$	$>10^5$
Ag/PZT/NSTO[17]	~ 1.2 nm	$>10^2$	3.5 V	$>10^5$	$>10^9$
Probe/BTO/NGO[18]	~ 1 nm	>2	3.5 V	---	---
W/HZO/SiO_2/Si[19]	~ 1 nm	$>10^2$	3 V	$>10^4$	$>10^3$
Au/Cr/BSO/NSTO (This Work)	~ 4.6 nm	$>10^9$	7 V	>10 years	$>5\times10^9$
	~ 2.1 nm	$>10^8$	6 V		
	~ 1 nm	$>7\times10^5$	4.5 V		

Supplementary References

1 Pantel, D. & Alexe, M. Electroresistance effects in ferroelectric tunnel barriers. *Phys. Rev. B* **82**, 134105 (2010).

2 Cuellar, F.A. et al. Thermally assisted tunneling transport in $La_{0.7}Ca_{0.3}MnO_3/SrTiO_3$: Nb Schottky-like heterojunctions. *Phys. Rev. B* **85**, 245122 (2012).

3 Barrett, J. H. Dielectric constant in perovskite type crystals. *Phys. Rev.* **86**, 118 (1951).

4 Sze, S. M. & Ng, K. K. in *Physics of Semiconductor Devices* 3rd edn (Wiley, 2007).

5 Zhang, S. et al. Giant polarization sustainability in ultrathin ferroelectric films stabilized by charge transfer. *Adv. Mater.* **29**, 1703543 (2017).

6 Shen, X-W. et al. Two-dimensional ferroelectric tunnel junction: the case of monolayer In:SnSe/SnSe/Sb:SnSe homostructure. *ACS Appl. Electron. Mater.* **1**, 1133-1140 (2019).

7 Mikheev, E., Hoskins, B. D., Strukov, D. B. & Stemmer, S. Resistive switching and its suppereson in Pt/Nb:$SrTiO_3$ junctions. *Nat. Commun.* **5**, 3990 (2014).

8 Card, H. C. & Rhoderick, E. H. Studies of tunnel MOS diodes I. Interface effects in silicon Schottky diodes. *J. Phys. D: Appl. Phys.* **4**, 1589 (1971).

9 Jerry, M. et al. Ferroelectric FET analog synapse for acceleration of deep neural network training. *International Electron Devices Meeting (IEDM)* 6.2.1-6.2.4. (IEEE, San Francisco, CA, USA, 2018).

10 Wu, J., Chen, HY., Yang, N. et al. High tunnelling electroresistance in a ferroelectric van der Waals heterojunction via giant barrier height modulation. *Nat. Electron.* **3**, 466–472 (2020).

11 Jin Hu, W., Wang, Z., Yu, W. et al. Optically controlled electroresistance and electrically controlled photovoltage in ferroelectric tunnel junctions. *Nat. Commun.* **7**, 10808 (2016).

12 Wen, Z., Li, C., Wu, D. et al. Ferroelectric-field-effect-enhanced electroresistance in metal/ferroelectric/semiconductor tunnel junctions. *Nat. Mater.* **12**, 617–621 (2013).

13 Li, J. et al. Giant electroresistance in ferroionic tunnel junctions. *IScience.* **16**, 368-377 (2019).

14 Ma, C., Luo, Z., Huang, W. et al. Sub-nanosecond memristor based on ferroelectric

tunnel junction. *Nat. Commun*. **11**, 1439 (2020).

15 Yang, Y. et al. Atomic-scale fatigue mechanism of ferroelectric tunnel junctions. *Sci. Adv.* **7**, eabh2716 (2021).

16 Xi, Z., Ruan, J., Li, C. et al. Giant tunnelling electroresistance in metal/ferroelectric/semiconductor tunnel junctions by engineering the Schottky barrier. *Nat. Commun*. **8**, 15217 (2017).

17 Luo, Z., Wang, Z., Guan, Z. et al. High-precision and linear weight updates by subnanosecond pulses in ferroelectric tunnel junction for neuro-inspired computing. *Nat. Commun*. **13**, 699 (2022).

18 Garcia, V., Fusil, S., Bouzehouane, K. et al. Giant tunnel electroresistance for non-destructive readout of ferroelectric states. *Nature* **460**, 81–84 (2009).

19 Cheema, S. et al. One nanometer HfO_2-based ferroelectric tunnel junctions on silicon. *Adv. Electron. Mat.* **8**, 2100499 (2022).
